# Innovation factor double circulation: Cross-border mobility and the manufacturing industry’s total factor productivity

**DOI:** 10.1371/journal.pone.0283693

**Published:** 2023-04-26

**Authors:** Wei Song, Jing He

**Affiliations:** School of Management of Xi`an University of Architecture and Technology, Xi`an, China; University of Cagliari: Universita degli Studi Di Cagliari, ITALY

## Abstract

**Objectives:**

The improvement of the manufacturing industry’s total factor productivity depends not only on innovation factor double circulation, but also on cross-border mobility to a large extent.

**Methodology:**

This paper constructs a model that demonstrates the impact of innovation factor double circulation and cross-border flow on the manufacturing industry’s total factor productivity, and it seeks to estimate this impact by using panel data from China’s manufacturing industry taken from the period 2009–2020.

**Findings:**

It finds the path dependence of innovation factors significantly increased their double circulation cost, and did not significantly improve the manufacturing industry’s total factor productivity.

**Conclusion:**

It finds the path dependence of innovation factors significantly increased their double circulation cost, and did not significantly improve the manufacturing industry’s total factor productivity. Cross-border flow improves the marginal efficiency of innovation factors, realizes the spatial agglomeration of high-end innovation factors and greatly promotes the double circulation of innovation factors in a way that effectively improves the manufacturing industry’s total factor productivity.

**Implications:**

These conclusions have profound policy implications: cross-border flows can promote the incremental adjustment of innovation factors; fully release the development potential and toughness of the dual circulation of innovation factors; and are essentially conducive to improving the manufacturing industry’s total factor productivity.

## 1 Introduction

It is widely acknowledged that manufacturing in a developing economic system provides an important way for innovative elements to use supply-side structural reform and open economic dividends that endow the innovation ecosystem with innovation and development resilience, expand double-cycle space and contribute to improvements in total factor productivity. In the first 30 years of Reform and Opening up, China’s manufacturing industry used large-caliber international circulation to realize the late-development and catch-up of technology, and it did this by applying the innovative mode of "learning by doing" and "learning by watching" during technology imitation and equipment introduction, which in turn laid a solid foundation for the manufacturing power strategy. As China’s manufacturing industry changes from a "runner who follow others" to a "runner abreast of others ", the "zigzag lead" between China and the United States has broken the previous US-led global value chain system. The "supply disconnection" of key core technologies and the "chain disconnection" of global industrial transfer have further increased the risk of anti-globalization, and the marginal contribution that uses international circulation to promote the technological ‘leap’ of China’s manufacturing industry is increasingly weak.

The improvement of the manufacturing industry’s total factor productivity in developing economies is framed against the background of trade frictions between major countries and depends on the extent to which the domestic and international double circulation of unimpeded innovation factors (subsequently the “double circulation of innovation factors”), regional invisible obstructions and institutional blocking points are eliminated. Cross-border flow is the internal driving force for optimizing institutional supply and realizing the double circulation of innovation factors [1]. After the 2010s, with the intensification of the anti-globalization trend and the rise of protectionism, the “double squeeze” of the international circulation demand side and the supply side has severely restricted the innovation and development space of China’s manufacturing industry, and as a result, the “choke” dilemma (caused by conflicts between major powers) has become an important incentive for inhibiting the improvement of the manufacturing industry’s total factor productivity [2]. As China’s manufacturing industry continues to expand towards both ends of the "smile curve", strengthening and expanding the regional and local effects of R&D spillovers has become the only way that China’s manufacturing industry can use R&D spillovers to achieve improved total factor productivity [3].

In this new domestic and international situation, the question of how to utilize the “internal and external promotion” of innovation factors to achieve continuous improvement in this respect has become an urgent problem for economists and policy makers to solve. The marginal contribution of this paper is that, it abandons the traditional idea of “studying the total factor productivity from the traditional international circulation”, and emphasizes how improvements of the cross-border flow caused by the double circulation of innovation factors impact manufacturing’s total factor productivity. This provides a new theoretical basis for explaining the effective improvement of the manufacturing total factor productivity that engages and incorporates the double circulation of innovation factors and cross-border flow in developing economies.

The first part of the paper puts forward the hypothesis that will be tested. The second part describes research samples, outlines data (and their processing mechanism), defines relevant variables, and constructs the econometric model; the third part uses panel data taken from China’s manufacturing industry in the period 2009–2020 to test the paper’s hypothesis, and carries out analysis and interprets estimated results; and the fourth part puts forward the paper’s conclusions and policy implications.

## 2 Literature and hypotheses

### 2.1 Literature review

The existing literature mainly offers three perspectives. First, a focus on how changes of innovation factor efficiency impact total factor productivity. Chen merges and unifies the classification and calibre of industrial industries, and uses panel data drawn from the period 1980–2008 to study changes of industrial total factor productivity and decomposition results that have occurred since the Reform and Opening-up were introduced. The results show the continuous improvement of total factor productivity is the fundamental symbol of the transformation of economic development mode, and also indicate that the replacement efficiency inculcated by the structural adjustment of innovative factors can significantly improve total factor productivity [4]. In drawing on this research contribution, Cai and Fu use the TFP Index Measurement Model to decompose the technical effects and structural effects of the 1978–2014 output and input data. They suggest the continuous elimination of backward production capacity and the construction of the reasonable incentive mechanism can, in operating in the wider context of supply-side structural reform, promote the aggregation of innovation factor resources in high-quality and efficient segmented industries and improve the allocation efficiency of innovation factor resources [5]. Liu et al. used the data of Chinese listed companies from 2003 to 2015 to study the impact of political links on innovation from the perspective of the heterogeneity of innovation quantity and quality. They found that political ties positively impact the amount of enterprise innovation, but are not conducive to innovation quality. Political ties weaken government subsidies for innovation quality and reduce enterprise R&D intensity. The government can improve enterprise innovation quality by improving marketization, and establishing intellectual property protection and anti-corruption measures [6].

Second, the improvement effect of different factors’ allocation quality on total factor productivity has been investigated by drawing on the quality difference of innovation factors. Hsieh and Klenow analyze industrial total factor productivity differences between China and the US by using micro-data drawn from industrial enterprises in the two countries. After controlling capital lease price, elasticity of factor substitution, policy influence and other variables, they find that 49% of the industrial total factor productivity difference between the two countries is caused by factor allocative efficiency [7]. On this basis, Wang and Niu estimate the industrial total factor productivity loss that occurred as a result of factor mismatch at the regional, industrial and overall industrial levels by using the data drawn from China’s industrial A-share listed companies in the period 2008–2017. They find factor mismatch degree in the eastern and central regions is low, and that it is highest in the manufacturing industry. If the factor mismatch degree is improved at the overall industry level, the total factor productivity of the country’s industry can be increased by 0.35–0.9 times [8]. Liu et al used data from Chinese listed companies in the period 2010–16 to investigate how two types of government R&D subsidies impacted innovation. They found that, when compared with post-reward, pre-reward has a better impact on innovation performance by stimulating private R&D investment. They also noted the effectiveness of government R&D subsidies weakened in enterprises with rent-seeking and political relations. This does not only provide a new perspective that can be used to understand the effect of government R&D subsidies, but also provides a point of reference that the government can use when seeking to improve public fund allocation efficiency [9].

Third, in drawing on a factor constraint perspective, this paper studies the influence of innovation factor bias type on total factor productivity. Acemoglu introduces endogenous and directed technological change constraints into the endogenous growth model to distinguish the factor bias of technological progress [10]. In further pursuing this research direction, Wang and Qi construct a transcendental logarithmic cost function model that includes R&D, international trade and foreign direct investment in a factor substitution framework, and also incorporates biased technological progress into it. After controlling for industrial differences, they find that the factor bias of technological progress from different sources determines the country’s industrial energy intensity and also note that in operating the factor substitution effect, energy saving technology progress can significantly reduce energy intensity [11]. Li and Li draw on the Kmenta Approximation Technique to derive the industrial total factor productivity function (including the biased technology progress index) from the standardized CES production function, and investigate the influence that biased technology progress has on the change of industrial total factor productivity, and also provide insight into its driving mechanism. They find that as the technological gap substantially reduces, the improvement effect of capital-biased technological progress on industrial total factor productivity weakens, which indicates long-term dependence on large-scale capital driving is not sustainable. In other words, in seeking to effectively improve industrial total factor productivity, actors should further optimize the rational allocation of factor input between industries [12].

### 2.2 Hypothesis proposition

It is well-established that the double circulation of innovation factor in developing economies exhibits the typical characteristics of path dependence. The manufacturing sector realizes the double circulation of innovation factor by introducing innovative factors that contain advanced knowledge and technology, and this is consistent with “learning by watching” and “learning by doing” [13]. It should be noted that the double circulation of innovation factor is often affected by various factors that include the scale of innovation, the home country’s technology sanctions and tariffs. In the double circulation of innovation factors process, developing economies usually choose a more effective double circulation path for improving production efficiency that includes cutting-edge technology intermediates, and this increases the cycle cost of innovation factors. When compared with more abundant and inexpensive localized innovation factors, the double circulation of innovation factors is more predisposed to use relatively abundant innovation factors to replace innovation factors with high cycle cost. The inconsistency of the cycle cost caused by the double circulation of innovation factor leads to the double circulation of innovation factor neglecting the improvement of innovation efficiency, and this leads to the double circulation of innovation factor to promote the inefficient concentration of relatively abundant factors. This orientates towards replacing the cycle efficiency in the unbalanced path, and inhibits the improvement of the manufacturing industry’s total factor productivity.

Hypothesis 1: The path dependence of innovation factors greatly increases their double circulation cost. It also causes the double circulation of innovation factors to accelerate the low efficiency agglomeration of relatively abundant factors along the unbalanced path. This has no significant effect on improvements in the manufacturing industry’s total factor productivity.

Previous studies show that the endogenous structure transformation mechanism, in operating under the new development pattern, can significantly promote improvements in the manufacturing industry’s total factor productivity [14]. The cross-border flow in the double circulation of innovation factor process breaks the institutional barrier of local protectionism to factor flow, greatly reduces the manufacturing industry’s innovation cost, improves the double circulation of innovation factor’s development potential, prolongs development toughness and improves the allocative efficiency of the double circulation of innovation factor. Through the cross-border flow of innovation factors, the double circulation continuously optimizes the initial endowment structure of innovation factors in the manufacturing industry. In operating under the action of factor price and marginal substitution, the marginal efficiency of innovation factors will greatly improve, and the spatial agglomeration of high-end innovation factors will be realized through cross-border flow. In other words, in a circumstance where the competitive selection mechanism reshapes innovation factors in the market, the double circulation of innovation factors achieves the agglomeration of high-end innovation factors in the high-efficiency manufacturing sector. It does this through a cross-border flow that forces the elimination of backward manufacturing capacity, and this in turn significantly improves the manufacturing industry’s total factor productivity.

Hypothesis 2: Cross-border flow improves the innovation factor’s marginal efficiency and this in turn realizes the high-end innovation factor’s spatial agglomeration, and greatly promotes effective improvements in the impact of the double circulation of innovation factor on manufacturing’s total factor productivity.

## 3 Research design

### 3.1 Measurement model

In order to estimate the impact of double circulation and cross-border flow of innovation factor on manufacturing industry total factor productivity, this paper constructs an econometric model whose expression is shown in Eq (1):

$$TFP_{i,t}=\alpha +\beta_{1}EDC_{i,t}+\beta_{1}EDC_{i,t}\cdot CBF_{i,t}+\gamma V_{i,t}+\varepsilon_{i,t}$$ (1)

In Eq (1), *TFP*_*i*,*t*_ represents the total factor productivity of manufacturing industry *i* in period *t*; *EDC*_*i*,*t*_ represents the double circulation of innovation factor of manufacturing industry *i* in period *t*; *CBF*_*i*,*t*_ represents the cross-border flow of manufacturing industry *i* in period *t*; *V*_*i*,*t*_ represents a set of control variables; and ε_*i*,*t*_ is the error term. In order to ensure the cross-section structure of the econometric model has a high degree of homogeneity, this paper uses the Forward Difference Method to eliminate the estimation deviation caused by the individual fixed effect, and this enables it to accurately reflect changes in the same cross-section at different time points.

### 3.2 Variable measurement

1. Manufacturing industry Total Factor Productivity (TFP). In order to sustain its ability to generalize, this paper uses the DEA-Malmquist index proposed by Färe et al. to estimate the manufacturing industry’s total factor productivity [15]. The details follow:
$$M\left(x_{t+1},\;y_{t+1},\;x_{t},\;y_{t}\right)={\left[\frac{D_{t}\left(x_{t+1},y_{t+1}\right)}{D_{\text{t}}\left(x_{t},y_{t}\right)}\cdot \frac{D_{t+1}\left(x_{t+1},y_{t+1}\right)}{D_{t+1}\left(x_{t},y_{t}\right)}\right]}^{\frac{1}{2}}$$ (2)
  In Eq (2),(*x*_*t*_,*y*_*t*+1_) represents the input-output vector in period t; (*x*_*t*+1_,*y*_*t*+1_) represents the input-output vector in period t+1; *D*_*t*_ represents the distance reference function in period t; and *D*_*t*+1_ represents the distance reference function in period t+1. In order to better explain the continuous growth of manufacturing TFP, this paper, in working on the basis of 2008, transforms the DEA-Malmquist TFP into the absolute TFP of manufacturing.
2. Innovation factor double circulation(EDC). The double circulation of innovation factors (EDC) is represented by the innovation factor flow in both domestic and international cycle systems. In this process, attraction is the main driving force behind the double circulation of innovation factors. This paper, therefore, includes attraction in the form of the EDC variable, which is described by referring to the gravity model:
$$EDC_{i,t}=g_{do,in}\cdot E_{do}^{g^{do}}\cdot E_{\dot{in}}^{g^{in}}\cdot D_{do,in}^{-d}.$$ (3)
  In Eq (3), *EDC*_*i*,*t*_ is the double circulation of innovation factors of manufacturing industry i in period t; *g*_*do*,*in*_ is the double circulation coefficient between domestic cycle (do) and international cycle (in); *E*_*do*_ and *E*_*in*_ are (respectively) the number of innovation factor used in domestic cycle (do) and international cycle (in); *g*^*do*^ and *g*^*in*^ are (respectively) the single cycle coefficient between innovation factor; *D*_*do*,*in*_ is the technical gap between domestic cycle (do) and international cycle (in); and *d* is the technical gap index (*d* = 2). In assuming cross-border flow of innovation factor with double circulation along the equilibrium path, the EDC index of innovation factor can be obtained by setting the value of circulation coefficient *g*_*do*,*in*_, *g*^*do*^ and *g*^*in*^ as 1, as shown in Eq (4):
$$EDC_{i,t}=E_{do}\cdot E_{in\;}\cdot D_{do,\;in\;}^{-2}$$ (4)
3. Cross-border flows (CBF). In following Huang and Ni, this paper measures cross-border flow (CBF) by using the intermediate product export rate (ERI) and final product import rate (FIR) [16]. The expression is shown in Eq (5):
$$CBF_{i,t}=\text{ln}\left(1+ERI_{i,t}\right)-\text{ln}\left(1+FIR_{i,t}\right)$$ (5)
  In Eq (5), the import rate of final goods(FIR) is the proportion of the total amount of final goods imported by foreign departments(IM)in the total amount of final goods used by THE COUNTRY’S manufacturing industry (FA), namely: *FIR*_*i*,*t*_ = *IM*_*i*,*t*_/*FA*_*i*,*t*_. Intermediate goods export rate (ERI) refers to the proportion of the total intermediate goods export (EX) of the country’s manufacturing industry in total intermediate goods production (PA), namely: *ERI*_*i*,*t*_ = *EX*_*i*,*t*_/*PA*_*i*,*t*_.
4. Control variables. In acknowledging that the influence of the double circulation of innovation factor on the total factor productivity of manufacturing is also affected by other variables, this paper seeks to ensure the accuracy of its results, this paper by introducing the degree of marketization (MAR), the degree of foreign trade (Tra) and the level of human capital (HUM) as its control variables. MAR measures the manufacturing innovation factor market’s sensitivity to changes in the supply and demand of manufacturing innovation products. This paper measures the proportion of the average number of those employed in non-state-owned enterprises above designated size in the manufacturing industry’s total average labor force. The degree of Foreign Trade (Tra) measures each manufacturing industry’s degree of openness. This paper uses proportion of export delivery value in each manufacturing industry’s main business income to measure it. Human capital level (HUM) is a measure of the quality of employed personnel in manufacturing industries. This paper uses the ratio of the full-time equivalent of each manufacturing industry’s R&D personnel to each manufacturing industry’s average employment to measure it.

### 3.3 Data sources

The sample data are unified into the preceding scale of the country’s manufacturing industry in the period 2009–2020; 2008 is taken as the constant price (2008 = 100). The variables, innovation factor double circulation (EDC), cross-border flow (CBF), degree of marketization (MAR) and human capital level (HUM) were taken from the China Industrial Statistical and China Industrial Economic Statistical yearbooks, and foreign trade degree (Tra) was derived from the China Import and Export Statistical and China Trade Statistical yearbooks. Missing data from the China Statistical Yearbook are addressed by using statistical methods published in each statistical yearbook over the course of the year. The descriptive statistical results of the main variables are shown in Table 1.

**Table 1:** Main variable descriptive statistics.

variable	Observed value	Mean value	Standard deviation	Minimum value	Maximum value
*TFP*	507	3.577	1.922	2.884	4.529
*EDC*	507	2.622	3.699	1.935	3.223
*CBF*	507	4.255	1.770	2.775	6.103
*MAR*	507	3.218	0.737	1.055	5.723
*TRA*	507	4.888	1.148	3.314	6.729
*HUM*	507	4.619	2.934	1.072	7.563

## 4 Results of econometric analysis

### 4.1 Basic estimation results

Table 2 lists estimation results obtained by Eq (1). Column (1) is the estimation result that includes the innovation factor double circulation as the explanatory variable; and Column (2) is the further estimation result that includes the cross-border flow variable.

**Table 2:** Basic estimation results: The influence of the double circulation and cross-border flow of innovation factors on the manufacturing industry’s total factor productivity.

variable	TFP	
	(1)	(2)
*EDC*	5.7011 (1.42)	—
*EDC×CBF*	—	6.1613** (2.07)
*MAR*	6.9654** (2.29)	4.8670*** (2.81)
*TRA*	2.6657* (1.95)	6.4247* (1.78)
*HUM*	5.0593* (1.83)	9.3065** (2.19)
*D_IND*	Yes	Yes
*D_TIM*	Yes	Yes
*Constant*	2.7415 (1.25)	3.0045 (1.37)
*F Statistics*	43.02	46.24
*Prob>F*	0.0000	0.0001
*R* ^*2*^	0.6471	0.6567
*Adj-R* ^*2*^	0.6294	0.6475

Note: (1) There is a t value in parentheses. (2) *, ** and *** respectively show that estimated parameter values are significant at the 10 percent, 5 percent and 1 percent confidence levels.

Column (1) of Table 2 shows every increase of EDC by 1 unit leads to an increase of 5.7011 units in the total factor productivity of manufacturing(This fails the significance test) and indicates the positive effect of EDC on the improvement of the manufacturing industry’s total factor productivity (TFP) is not significant. This paper argues that the traditional path dependence on the manufacturing industry’s double circulation of innovation factor results in the industry’s technological progress being suppressed by technology frontier exporters, and this weakens the play of the double circulation of innovation factor. The technology spillover created by innovation factors benefits from participation in the global value chain division of labor and improves the total factor productivity of China’s manufacturing industry to a certain extent. It also creates more fierce market competition for China’s manufacturing industry and puts forward higher requirements for domestic technology absorption. As the manufacturing industry labor costs rises, the strengthening of technical barriers in developed countries further squeezes the industry’s living space, and the difficulties associated with absorbing advanced technology further increase. And the resulting competitive effect weakens the promotion of the double circulation of innovation factors in the industry’s total factor productivity. And the increasing cost of high-tech intermediate products in China’s manufacturing industry simultaneously inhibits the effective allocation of the double circulation of innovation factor to the intermediate product market. The substitution effect of innovation factor with high cycle cost on relatively abundant innovation factors is not obvious, and the double circulation of innovation factor and the market allocation tendency of innovation factor have typically inconsistent characteristics, which in turn suggests that the path dependence of innovation factors can greatly improve the double circulation cost of innovation factor, and makes it difficult for China’s manufacturing industry to achieve resource integration and fundamental breakthroughs in technology. In this sense, the double circulation of innovation factor cannot significantly improve the manufacturing industry’s total factor productivity of manufacturing industry. On this basis, Hypothesis 1 is accepted.

Column (2) of Table 2 shows every 1 unit increase of the interaction term (EDC×CBF) of the double circulation of innovation factor and cross-border flow leads to a 6.1613 unit increase in the manufacturing industry’s total factor productivity (TFP), and this result is shown to be significant at the 5 percent confidence level. The results show innovation factor double circulation (EDC) and cross-border flow (CBF) have a significant positive effect on the improvement of the manufacturing industry’s total factor productivity (TFP). One reasonable explanation is that the unimpeded cross-border flow prevents innovation factors between regions from being obstructed. As the initial innovation factor endowment structure in the country’s manufacturing industry is optimized, the consistency of matching innovation factor supply and innovation factor demand greatly promotes improvements in the double-circulation blocking point of innovation factor. In this process, the significant increase in the complexity of manufacturing production technology has further promoted the independent research and development of core technology and disrupted technological innovation. It has also accelerated the independent incubation of intelligent manufacturing technology, and led to significant development of R&D spillover effects through joint development of domestic R&D and international R&D. The absorption and diffusion of high and new technologies such as big data stimulate the traditional manufacturing industry’s rapid transition from labor-intensive to technology-intensive and capital-intensive. They also promote the added value of China’s manufacturing products, and drive its manufacturing industry forward in the global value chain. They also improve the substitution elasticity and technical complexity of the country’s manufacturing production elements, and this in turn promotes the improvement of manufacturing’s total factor productivity. In other words, in the double circulation of innovation factor process, the stimulation of innovation vitality promotes spatial agglomeration and enables innovation factors in manufacturing industry sectors to be upgraded, and this in turn provides an internal driving force that generates improvements in the manufacturing industry’s total factor productivity. In this sense, cross-border flow (CBF) significantly improves the positive effect of innovation factor double cycle (EDC) on the total factor productivity manufacturing industry (TFP). On this basis, Hypothesis 2 is accepted.

### 4.2 Robustness estimation results

The production rate of new products (OVNP) in the manufacturing industry is the relative marginal output created by the continuous spatial agglomeration of high-end innovation factor in the double circulation of innovation factor process. It reflects the comprehensive efficiency change of the double circulation of innovation factor, which is reflected in the direct contribution of the double circulation of innovation factor to new product output. This paper uses the value of new products in manufacturing as a substitute variable for total factor productivity to conduct a robustness test. The estimated results are shown in Table 3.

**Table 3:** Robustness estimation results: The influence of innovation factor double circulation and cross-border flow on the productivity of new manufacturing products.

variable	OVNP	
	(1)	(2)
*EDC*	2.1032 (1.41)	—
*EDC×CBF*	—	4.1504** (2.25)
*MAR*	4.1721** (2.09)	2.0497*** (2.76)
*TRA*	2.1663* (1.78)	5.5403* (1.69)
*HUM*	3.5271** (2.48)	7.5647** (2.41)
*D_IND*	Yes	Yes
*D_TIM*	Yes	Yes
*Constant*	1.8456 (1.17)	1.4965 (1.36)
*F Statistics*	40.20	45.88
*Prob>F*	0.0000	0.0000
*R* ^*2*^	0.6684	0.6922
*Adj-R* ^*2*^	0.6517	0.6856

Note: (1) t value is indicated in parentheses. (2) *, ** and *** respectively show that estimated parameter values are significant at the 10%, 5% and 1% confidence levels.

Column (1) and (2) of Table 3 show that the robust estimation results that apply innovation factor double circulation (EDC) and the interaction term between innovation factor double circulation and cross-border flow (EDC×CBF) as explanatory variables are not significantly different to the parameters explained by the manufacturing new product value rate, and this indicates that the econometric model setting and the basic estimation results are highly robust.

### 4.3 Endogenous estimation results

The double circulation of innovation factor (EDC) embodies the cycle of production, distribution, exchange and consumption of innovation factor in domestic and international markets. As anti-globalization intensifies, the double circulation of innovation factor manifests as the mutual promotion and unification of the blocking point of international circulation breakthrough and the smooth circulation of domestic circulation. This feature is manifested by the level of external dependence (DEP). In this paper, external dependency (DEP) is used as an instrumental variable of innovation factor double circulation (EDC) by the endogenous test. The estimated results are shown in Table 4.

**Table 4:** Endogenous estimation results: The influence of innovation factor double circulation and cross-border flow on manufacturing industry total factor productivity.

variable	*TFP*	
	(1)	(2)
*DEP*	1.0547 (1.37)	—
*DEP*×*CBF*	—	3.7950** (2.24)
*MAR*	3.2896** (2.00)	1.9935*** (2.97)
*TRA*	2.0672* (1.69)	4.6826* (1.86)
*HUM*	2.9364** (2.32)	6.8319** (2.43)
*D_IND*	*Yes*	*Yes*
*D_TIM*	*Yes*	*Yes*
*Constant*	1.792 (1.25)	1.3862 (1.14)
*F Statistics*	40.19	45.88
*Prob*>*F*	0.0001	0.0003
*R* ^2^	0.6724	0.692
*Adj-R* ^2^	0.6637	0.685

Note: (1) t value indicated in parentheses. (2) *, ** and *** respectively represent estimated parameter values are significant at the 10 percent, 5 percent and 1 percent confidence levels.

Column (1) and (2) of Table 4 show that the endogenous estimation results with external dependency (DEP) and interaction term between external dependency and cross-border flow (DEP×CBF), when considered as explanatory variables, generally show a downward trend in the explanatory coefficient of the manufacturing industry’s total factor productivity (TFP). Generation does not change significantly, which weakens the model endogeneity. This shows the econometric model’s setting is reasonable.

## 5 Conclusions and policy implications

### 5.1 Research conclusion

This paper constructs a model that provides insight into the influence of the double circulation of innovation factor and cross-border flow on the manufacturing industry’s total factor productivity, and it achieves this by drawing on panel data drawn from China’s manufacturing industry in the period 2009–2020. After controlling the influence of the degree of marketization, the degree of foreign trade and the level of human capital, it finds the innovation factor’s path dependence greatly increases this factor’s double circulation cost. The double circulation of innovation factor accelerates the low efficiency agglomeration of relatively abundant factors along the unbalanced path, and does not significantly affect the improvement of the manufacturing industry’s total factor productivity. Cross-border flow improves the marginal efficiency of innovation factor, realizes the spatial agglomeration of the high-end innovation factor, and greatly promotes the effective improvement of the impact of the double circulation of innovation factor on the manufacturing industry’s total factor productivity.

### 5.2 Policy implications

These conclusions have profound policy implications. First, the development potential and resilience of the double circulation of innovation factor should be fully released to force the manufacturing industry to significantly improve its total factor productivity by operationalizing the high-quality incremental innovation factor. We will remove organizational barriers that prevent the double circulation of innovation factor, unblock the flow of innovation factor and promote the independent and orderly flow of innovation factor at both domestic and international levels. Strengthen the top-level mechanism design of the double circulation of innovation factor and overall planning allocation; improve the sequential growth mechanism of the double circulation of innovation factor; and improve the leading and supporting role that the double circulation of innovation factor performs in the manufacturing industry’s total factor productivity. Particular attention should be paid to assisting the spatial agglomeration of innovation factor to transform low-efficiency sectors. In addition, efforts should also be made to optimize the double-circulation transmission path of innovation factor, and realize the effective allocation and utilization of innovation factor–both innovations will in turn enable the manufacturing industry to achieve high-quality development.

Second, the cross-border flow mechanism of double circulation of innovation factor should be further strengthened; the incremental adjustment of innovation factor should be driven by the optimization of innovation factor stock; and the incremental disadvantage of low efficiency should be transformed into the advantage of high efficiency stock by relying on cross-border flow, which in turn help to substantially improve the manufacturing industry’s total factor productivity. This will promote the continuous enhancement of the accumulation of innovation factor in China’s manufacturing industry; improve the substitution role of innovation factor relative to other factors; and help to realize the dynamic upgrading of innovation factor endowment structure, and in turn establish a comparative advantage in the intensive use of the innovation factor. We should also improve the market allocation mechanism for cross-border mobility, remove institutional and organizational barriers to cross-border mobility, and strengthen the role of cross-border mobility in the allocation of innovation factor. We should further innovate in developing the market mechanism of double-circulation and cross-border flow, weaken the reaction time delay of cross-border innovation factor flow, greatly improve the effectiveness of market allocation of the innovation factor, enhance the suitability of the double circulation of the innovation factor, and improve the ability of the double circulation of the innovation factor to promote the growth of the manufacturing industry’s total factor productivity.

In referring to the traditional double circulation of innovation factors that affect the manufacturing industry’s total factor productivity, this paper further incorporates cross-border flows into its research framework and examines the effect that the double circulation of innovation factors and cross-border flow have on the industry’s total factor productivity, and also provides a point of reference for ongoing efforts to improve the industry’s total factor productivity. It should be noted that the lack of in-depth investigation of other industries makes it difficult to transfer the research conclusions to them. In any case, any such comparison is complicated by differences in industry technological maturity conditions and resource endowments, along with different development stages, which make it difficult to make a horizontal comparison across individual industries. The field survey method should be applied and real research materials should be used, as this will help to establish a more objective research framework that will benefit further research.

## Supporting information

S1 File (XLS)

S2 File (XLS)

## References

[1] Hu Y. The political economic research on Sino-US trade friction. Foreign Economic Relations & Trade. 2019; (4): 6–12.

[2] Wang B Y. High-quality development of innovative manufacturing industry: Characteristic facts, drivers and supporting factors. China Soft Science. 2021; (10):148–159.

[3] Dong J R, Zhang W Q. Technology source, technology progress bias and China"s manufacturing upgrading——thoughts on the dual circulation new development pattern. Forum on Science and Technology in China. 2021; (10): 71–82.

[4] Chen S Y. Reconstruction of sub-industrial statistical data in China: 1980–2008. China Economic Quarterly. 2011; (3): 735–776.

[5] Cai Y Z, Fu Y F. The technical and structural effects of TFP growth: measurement and decomposition based on China’s Macro and sector data. Economic Research. 2017; (1): 72–88.

[6] Liu S, Du J, Zhang W, Tian X, Kou G. Innovation quantity or quality? the role of political connections. Emerging Markets Review. 2021; (9): 1–29.

[7] Hsieh C T, Klenow P J. Misallocation and Manufacturing TFP in China and Indian. Quarterly Journal of Economics. 2009; (4): 1403–1448.

[8] Wang W, Niu Z D. Resources misallocation and multi-dimensional industrial TFP in China. The Journal of Quantitative & Technical Economics. 2019; (3): 20–37.

[9] Liu S, Du J, Zhang W, Tian X. Opening The Box of Subsidies: Which is More Effective for Innovation?[J]. Eurasian Economic Review, 2021, (11): 421–449.

[10] Acemoglu D. Directed Technical. The Review of Economic Studies. 2002; (4): 781–809.

[11] Wang B B, Qi S Z. Biased technological progress, factor substitution and China’s Industrial energy intensity. Economic Research. 2014; (2): 115–127.

[12] Li X P, Li X K. Biased Technological Change and the Total Factor Productivity Growth of China’s Industry. Economic Research, 2018; (10): 82–96.

[13] He S. Growing Through endogenous innovation cycles. Journal of Macroeconomics. 2022; (71): 1–12.

[14] Huang J, Wei J. Impact of intelligent development on the total factor productivity of firms—based on the evidence from listed Chinese manufacturing firms. Journal of Advanced Computatioanl Intelligence and Intelligent Informatics. 2022; (4): 555–561.

[15] Färe R, Grosskopf S, Norris M, Zhang Z. Productivity growth, technical progress, and efficiency change in industrialized countries. The American Economic Review. 1994; (1): 66–83.

[16] Huang Q H, Ni H F. Measurement of domestic and international double cycle of China’s economy: The essential characteristics of the new development pattern. Management World. 2021; (12): 40–58.

